# M. Ricord on Cholera

**Published:** 1885-03

**Authors:** 


### M. Ricord on Cholera.

M. Ricord, at a recent meeting of the Académie de Médecine, made what he described as a confession of faith. He had witnessed all the epidemics of cholera, the first, in 1832, included. At that moment there were six hundred patients at the hospital when he was physician. Not one of these six hundred either before the entry or during the residence of cholera patients, was attacked with cholera. Not one of the nurses, male or female, nor of the medical officers, caught cholera. Dr. Ricord adds that during this epidemic he did not see any facts which would leave him to believe the cholera is contagious. His conviction remains the same after studying the subsequent epidemics, and he is strongly opposed to quarantines, which are irksome and useless.

